# Clinical characteristics and outcome of influenza virus infection among adults hospitalized with severe COVID-19: a retrospective cohort study from Wuhan, China

**DOI:** 10.1186/s12879-021-05975-2

**Published:** 2021-04-12

**Authors:** Xunliang Tong, Xiaomao Xu, Guoyue Lv, He Wang, Anqi Cheng, Dingyi Wang, Guohui Fan, Yue Zhang, Yanming Li

**Affiliations:** 1 Department of Pulmonary and Critical Care Medicine, Beijing Hospital, National Center of Gerontology, the Institute of Geriatric Medicine, Chinese Academy of Medical Sciences, Beijing, People’s Republic of China; 2 First Department of Hepatobiliary & Pancreas Surgery, The First Hospital, Jilin University, Jilin, 130021 Changchun China; 3 Tobacco Medicine and Tobacco Cessation Center, Center of Respiratory Medicine, China-Japan Friendship Hospital, Beijing, China; 4 WHO Collaborating Center for Tobacco Cessation and Respiratory Diseases Prevention, Beijing, China; 5 National Clinical Research Center for Respiratory Diseases, Beijing, China; 6 Institute of Respiratory Medicine, Chinese Academy of Medical Sciences, Beijing, China; 7 Institute of Clinical Medical Sciences, China-Japan Friendship Hospital, Beijing, China; 8 Institute of Respiratory Medicine, Chinses Academy of Medical Sciences; National Clinical Research Center for Respiratory Disease, Beijing, China

**Keywords:** COVID-19, Influenza virus IgM, SARS-CoV-2

## Abstract

**Background:**

Coronavirus disease 2019 (COVID-19) is an emerging infectious disease that rapidly spreads worldwide and co-infection of COVID-19 and influenza may occur in some cases. We aimed to describe clinical features and outcomes of severe COVID-19 patients with co-infection of influenza virus.

**Methods:**

Retrospective cohort study was performed and a total of 140 patients with severe COVID-19 were enrolled in designated wards of Sino-French New City Branch of Tongji Hospital between Feb 8th and March 15th in Wuhan city, Hubei province, China. The demographic, clinical features, laboratory indices, treatment and outcomes of these patients were collected.

**Results:**

Of 140 severe COVID-19 hospitalized patients, including 73 patients (52.14%) with median age 62 years were influenza virus IgM-positive and 67 patients (47.86%) with median age 66 years were influenza virus IgM-negative. 76 (54.4%) of severe COVID-19 patients were males. Chronic comorbidities consisting mainly of hypertension (45.3%), diabetes (15.8%), chronic respiratory disease (7.2%), cardiovascular disease (5.8%), malignancy (4.3%) and chronic kidney disease (2.2%). Clinical features, including fever (≥38 °C), chill, cough, chest pain, dyspnea, diarrhea and fatigue or myalgia were collected. Fatigue or myalgia was less found in COVID-19 patients with IgM-positive (33.3% vs 50/7%, *P* = 0.0375). Higher proportion of prolonged activated partial thromboplastin time (APTT) > 42 s was observed in COVID-19 patients with influenza virus IgM-negative (43.8% vs 23.6%, *P* = 0.0127). Severe COVID-19 Patients with influenza virus IgM positive have a higher cumulative survivor rate than that of patients with influenza virus IgM negative (Log-rank *P* = 0.0308). Considering age is a potential confounding variable, difference in age was adjusted between different influenza virus IgM status groups, the HR was 0.29 (95% CI, 0.081–1.100). Similarly, difference in gender was adjusted as above, the HR was 0.262 (95% CI, 0.072–0.952) in the COX regression model.

**Conclusions:**

Influenza virus IgM positive may be associated with decreasing in-hospital death.

**Supplementary Information:**

The online version contains supplementary material available at 10.1186/s12879-021-05975-2.

## Background

In December 2019, a novel coronavirus with high similarity to the coronavirus responsible for severe acute respiratory syndrome (SARS-CoV) appeared and was later named as SARS-CoV-2 [1–3]. In 2020, the World Health Organization (WHO) announced that the pandemic of Coronavirus disease 2019 (COVID-19) has constituted a public health emergency of international concern [4].

Previous studies have focused primarily on COVID-19 patients’ clinical features with fever accompanied with respiratory and/or gastrointestinal symptoms, and so on [5, 6], which were highly similar to the clinical manifestation of influenza like illness (ILI). ILI may occur in population as co-infection of SARS-CoV-2 and influenza virus during the pandemic. Sustained surveillance of ILI has been implemented by Centers for Disease Control and Prevention (CDC) [7–9] and the co-infection of influenza viruses and SARS-CoV-2 was possible at 2019–2020 influenza season [2, 10]. According to previous study, influenza virus-specific antibody responses following influenza infection rises in HA-specific serum IgM (86 to 94%) antibodies after primary influenza virus infection in adults [11]. Therefore, HA-specific serum IgM can be identified as the marker of influenza virus infection in COVID-19 patients. The aims of this study were to describe the clinical features and outcomes of hospitalized COVID-19 patients, who were also positive of influenza virus IgM.

## Methods

### Study design

**Fig. 1:**
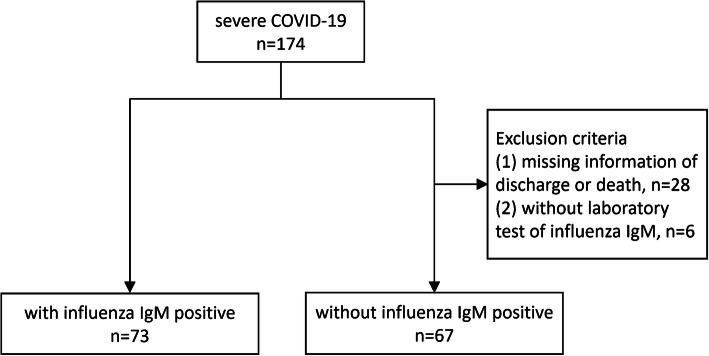
Flowchart

This was a retrospective cohort study which was performed during Feb 8th to March 15th at wards designated for patients with COVID-19 in the Sino-French New City Branch of Tongji Hospital in Wuhan city, Hubei province, China. Total of 140 patients diagnosed of COVID-19 pneumonia was enrolled from two wards managed by multidisciplinary team from Beijing Hospital and First Hospital attached to Jilin University (Fig. 1). The study was approved by Ethics Committee of Beijing Hospital (2020BJYYEC-046-01).

The inclusion criteria: throat-swab specimen from upper respiratory tract that were obtained and tested by RT-PCR for confirmation of SARS-CoV-2 as the same protocol described previously [1, 12]; pneumonia confirmed by thoracic CT scan [13], an oxygen saturation (SaO2) of 94% or less while they were breathing ambient air or a ratio of the partial pressure of oxygen (PaO2) to the fraction of inspired oxygen (FiO2) at or below 300 mmHg [14]. Exclusion criteria included without examined influenza virus IgM in the first 24 h in hospital, sudden death within 24 h.

### Data collection

All the data from electronic medical records were reviewed by experienced physicians separately and checked by 2 physicians independently. The baseline of clinical data was recorded in the first 24 h after administration and all interventions and the highest level of oxygen support during hospitalization were recorded.

Throat swab samples were collected for SARS-CoV-2 detection from patients by local Centers for Disease Control and Prevention, local health institutions. The PCR re-examination was conducted by throat-swab specimens after clinical remission of symptoms, including fever, cough, and dyspnea. A patient was allowed to discharge if he was clinical improvement and two throat-swab samples negative for SARS-CoV-2 RNA obtained at least 24 h apart [14]. Peripheral blood samples from patients were taken for identification of influenza virus-specific antibody IgM which responses following influenza infection and detected by indirect immunofluorescence assay (Respiratory tract 8 joint detection kit; EUROIMMUN, Inc., Germany) [11, 15, 16].

### Statistical analysis

Descriptive analyses of the variables were expressed as median (interquartile range [IQR]) or number (%) and compared using Mann-Whitney test. Categorical data were compared using *X*^2^ test or the Fisher exact test, where appropriate. The patients’ characteristics of deaths verses discharged and death/discharge & influenza IgM positive/negative were also described and shown in Supplementary Table 1 and 2. Kaplan-Meier curve was portrayed by influenza virus IgM positive/negative to describe the cumulative survival rate of COVID-19 patients. COX regression model was fitted to investigate the association between influenza virus IgM positive and the in-hospital death. To avoid overfitting, at most two covariates were allowed to the model and we adjusted for age and gender respectively in the model. Adjusted hazard ratios (aHRs) and 95% confidence intervals (95% CIs) were then estimated. All tests were 2-sides, and a *P* value less than .05 was considered statistically significant. All analyses were performed with SPSS, version 23.0 (IBM inc.).

## Results

### Baseline characteristics

A total of 140 adult patients confirmed with COVID-19 from designated hospital was enrolled in this study, with 73 patients (52.14%) were identified as influenza virus IgM-positive. 76 (54.4%) of the COVID-19 patients were males. The median age of patients with influenza virus-IgM negative was 66 years (IQR, 55 to 70 years), older than patients with influenza virus IgM-positive (median age 62, IQR, 47 to 70 years, *P* = 0.1118). Chronic comorbidities consisting mainly of hypertension (45.3%), diabetes (15.8%), chronic respiratory disease (7.2%), cardiovascular disease (5.8%), malignancy (4.3%) and chronic kidney disease (2.2%). Clinical features, including fever (≥38 °C), chill, cough, chest pain, dyspnea, diarrhea and fatigue or myalgia were collected. Fatigue or myalgia was less found in COVID-19 patients with IgM-positive (33.3% vs 50/7%, *P* = 0.0375). (Table 1).

**Table 1:** Clinical Characteristics of COVID-19 Patients with and Without Influenza IgM positive

Study Population	With Influenza IgM positive (***n*** = 73)	Without Influenza IgM positive (***n*** = 67)	Total (***n*** = 140)	***P*** value
**Demographic**				
Gender, Male	39 (53.4)	37 (55.2)	76 (54.3)	0.8310
Age, media (IQR), yrs	62.0 (47.0, 70.0)	66.0 (55.0, 70.0)	65.0 (48.5, 70.0)	0.1118
Comorbidities				
Hypertension	32/70 (45.7)	30/67 (44.8)	62/137 (45.3)	0.9122
Diabetes	12/72 (16.7)	10/67 (14.9)	22/139 (15.8)	0.7787
Chronic respiratory disease	5/72 (6.9)	5/67 (7.5)	10/139 (7.2)	0.9060
Cardiovascular disease	5/72 (6.9)	3/67 (4.5)	8/139 (5.8)	0.5301
Malignancy	3/72 (4.2)	3/67 (4.5)	6/139 (4.3)	0.9282
Chronic kidney disease	2/72 (2.8)	1/67 (1.5)	3/139 (2.2)	0.5983
**Signs and symptoms**				
Fever	55 (75.3)	53 (79.1)	108 (77.1)	0.5964
Highest temperature, °C	38.5 (38.0, 39.0)	38.7 (38.2, 39.0)	38.5 (38.0, 39.0)	0.1274
Chills	13 (17.8)	19 (28.4)	32 (22.9)	0.1375
Cough	41/72 (56.9)	44/67 (65.7)	85/139 (61.2)	0.2915
Productive cough	20/72 (27.8)	25/67 (37.3)	45/139 (32.4)	0.2299
Chest pain/Chest congestion	19/72 (26.4)	13/67 (19.4)	32/139 (23.0)	0.3283
Dyspnea	21/72 (29.2)	29/67 (43.3)	50/139 (36.0)	0.0831
Diarrhea	18 (24.7)	25 (37.3)	43 (30.7)	0.1049
Fatigue or myalgia	24/72 (33.3)	34/67 (50.7)	58/139 (41.7)	0.0375
**Laboratory findings, median (IQR)**				
White blood cells, × 10^9^/mL	5.7 (4.2, 6.8)	5.7 (4.6, 7.9)	5.7 (4.4, 7.2)	0.3226
Neutrophils, × 10^9^/mL	3.9 (2.5, 4.8)	4.0 (2.6, 5.9)	3.9 (2.5, 5.3)	0.3600
Lymphocytes, ×10^9^/mL	1.2 (0.9, 1.6)	1.1 (0.8, 1.5)	1.1 (0.8, 1.5)	0.3826
Lymphocytes< 0.8 × 10^9^/mL	18/73 (24.7)	18/66 (27.3)	36/139 (25.9)	0.7252
Red blood cells, × 10^12^/mL	4.1 (3.6, 4.6)	4.0 (3.7, 4.4)	4.0 (3.7, 4.5)	0.4502
Platelets, ×10^9^/ mL	230.0 (173.0, 292.0)	253.0 (169.0, 340.0)	235.0 (169.0, 312.0)	0.3622
Platelets< 100 × 10^9^/mL	5/73 (6.8)	6/66 (9.1)	11/139 (7.9)	0.6249
Hemoglobin, g/L	122.0 (114.0, 137.0)	125.5 (113.0, 137.0)	123.0 (113.0, 137.0)	0.9143
ALT, U/L	23.0 (17.0, 40.0)	22.5 (15.0, 41.0)	23.0 (16.0, 41.0)	0.7373
AST, U/L	26.0 (19.0, 37.0)	30.0 (19.0, 41.0)	28.0 (19.0, 39.0)	0.3370
Albumin, g/L	36.1 (32.2, 38.3)	35.0 (31.4, 37.1)	35.2 (31.7, 38.1)	0.2945
Creatinine, μmol/L	70.0 (60.0, 89.5)	70.0 (59.0, 87.0)	70.0 (59.0, 89.0)	0.8596
LDH, U/L	268.5 (204.0, 329.5)	287.0 (235.0, 351.0)	281.0 (212.0, 334.0)	0.2419
LDH > 245 U/L	44/72 (61.1)	47/65 (72.3)	91/137 (66.4)	0.1658
Troponin> 15.6 pg/mL, No (%)	7/51 (13.7)	12/56 (21.4)	19/107 (17.8)	0.2977
NT-proBNP, pg/mL	140.0 (60.0, 334.0)	157.0 (64.0, 459.0)	151.0 (63.0, 411.0)	0.2883
NT-proBNP≥247 pg/mL, No (%)	29/57 (50.9)	35/58 (60.3)	64/115 (55.7)	0.3069
CRP, mg/L	21.3 (4.1, 49.2)	34.7 (9.1, 73.4)	27.2 (6.1, 69.8)	0.1281
CRP ≥ 1 mg/L, No (%)	57/61 (93.4)	47/49 (95.9)	104/110 (94.5)	0.5651
IL-6, pg/mL	9.8 (4.2, 21.1)	6.8 (3.6, 23.2)	9.4 (3.9, 23.2)	0.5603
IL-6 ≥ 7 pg/mL, No (%)	25/42 (59.5)	15/35 (42.9)	40/77 (51.9)	0.1450
Ferritin, μg/L	522.1 (320.5, 729.0)	630.5 (310.2, 1519.9)	562.6 (320.5, 986.5)	0.0964
Ferritin> 150 μg/L, No (%)	39/43 (90.7)	33/35 (94.3)	72/78 (92.3)	0.5495
PT, s	13.7 (13.2, 14.3)	13.8 (13.4, 14.2)	13.8 (13.3, 14.3)	0.9762
APTT, s	39.6 (35.8, 42.0)	39.5 (37.8, 45.8)	39.6 (36.6, 44.3)	0.0243
APTT> 42 s, No (%)	17/72 (23.6)	28/64 (43.8)	45/136 (33.1)	0.0127
FIB, g/L	4.9 (3.9, 6.0)	5.3 (4.3, 6.2)	5.0 (4.1, 6.1)	0.2374
D-Dimer, μg/mL	0.7 (0.5, 1.7)	1.2 (0.5, 2.1)	1.0 (0.5, 2.0)	0.2371
D-Dimer≥0.5 μg/mL, No (%)	53/73 (72.6)	45/64 (70.3)	98/137 (71.5)	0.7669

Note. Data are presented as n (%) or median (IQR, interquartile range) for each parameter. *P* values were calculated by chi-square test, Fisher’s exact test, or Mann-Whitney U test, where appropriate

*Abbreviations IQR* interquartile range, *ALT* alanine aminotransferase, *AST* aspartate aminotransferase, *LDH* lactic Acid dehydrogenase, *CRP* C-reactive protein, *IL-6* interleukin-6, *PT* prothrombintime, *APTT* activated partial thromboplastin time, *FIB* fibrinogen

### Laboratory findings

Higher proportion of prolonged activated partial thromboplastin time (APTT) > 42 s was observed in COVID-19 patients with influenza virus IgM-negative (43.8% vs 23.6%, *P* = 0.0127). (Table 1) Counts of lymphocytes and platelets were significantly lower, while aspartate aminotransferase (AST), creatinine, lactate dehydrogenase (LDH), troponin, NT-proBNP, C-reactive protein (CRP), interleukin-6 (IL-6), ferritin, prothrombin time (PT), APTT and D-Dimer were significantly higher in dead cases (all *P* < 0.05). (Supplementary Table 1).

### Treatment and outcomes

43.6% of the patients received nasal cannula, 2.1% oxygen mask, 49.3% non-invasive mechanical ventilation (NMV)/high-flow nasal cannula (HFNC) and 8.6% invasive mechanical ventilation (IMV)/extracorporeal membrane oxygenation (ECMO). Compound Methoxamine capsule were used in more patients with influenza IgM positive than the other group (23.3% vs 9.0%, *P* = 0.0222). (Table 2) higher levels of respiratory support were more seen in dead patients, especially those with influenza IgM positive. (supplementary Table 1 and supplementary Table 2)

**Table 2:** Treatment and prognosis of COVID-19 Patients with and Without Influenza IgM positive

Study Population	With influenza IgM positive (***n*** = 73)	Without influenza IgM positive (***n*** = 67)	Total (***n*** = 140)	***P*** value
Treatment in hospital				
Oxygen Therapy				
Nasal Cannula	32 (43.8)	29 (43.3)	61 (43.6)	0.9475
Oxygen Mask	1 (1.4)	2 (3.0)	3 (2.1)	0.5069
NMV/High-flow nasal cannula	35 (47.9)	34 (50.7)	69 (49.3)	0.7405
IMV/ECMO	7 (9.6)	5 (7.5)	12 (8.6)	0.6535
Drugs				
Oseltamivir	33 (45.2)	23 (34.3)	56 (40.0)	0.1894
Arbidol	53 (72.6)	47 (70.1)	100 (71.4)	0.7482
Compound Methoxamine capsule	17 (23.3)	6 (9.0)	23 (16.4)	0.0222
Clinical outcomes				
CURB-65				0.0397
Low risk	65 (89.0)	50 (74.6)	115 (82.1)	
Medium risk	6 (8.2)	8 (11.9)	14 (10.0)	
High risk	2 (2.7)	9 (13.4)	11 (7.9)	
Duration of viral shedding, days	26.0 (20.0, 32.0)	25.0 (21.0, 32.0)	25.5 (20.5, 32.0)	0.9694
Hospital length of stay, days	13.0 (10.0, 18.0)	14.0 (10.0, 17.0)	13.0 (10.0, 18.0)	0.9084
Time from illness onset to discharge, days	27.0 (22.0, 35.0)	27.0 (21.0, 33.0)	27.0 (22.0, 33.5)	0.6208
Death,No (%)				0.0276
Discharge	70 (95.9)	57 (85.1)	127 (90.7)	
Death	3 (4.1)	10 (14.9)	13 (9.3)	

According to the score of CURB-65, more COVID-19 patients with influenza IgM positive group were in low to moderate risk level (*P* = 0.0397). No differences were observed in the duration of viral shedding, the length of hospital stay and time from illness onset to discharge between groups. 9.3% of the patients died in hospital and the rate of death was significantly lower in patients with IgM positive than those with IgM negative (4.1% vs 14.9%, *P = 0.0276*). (Table 2).

**Fig. 2:**
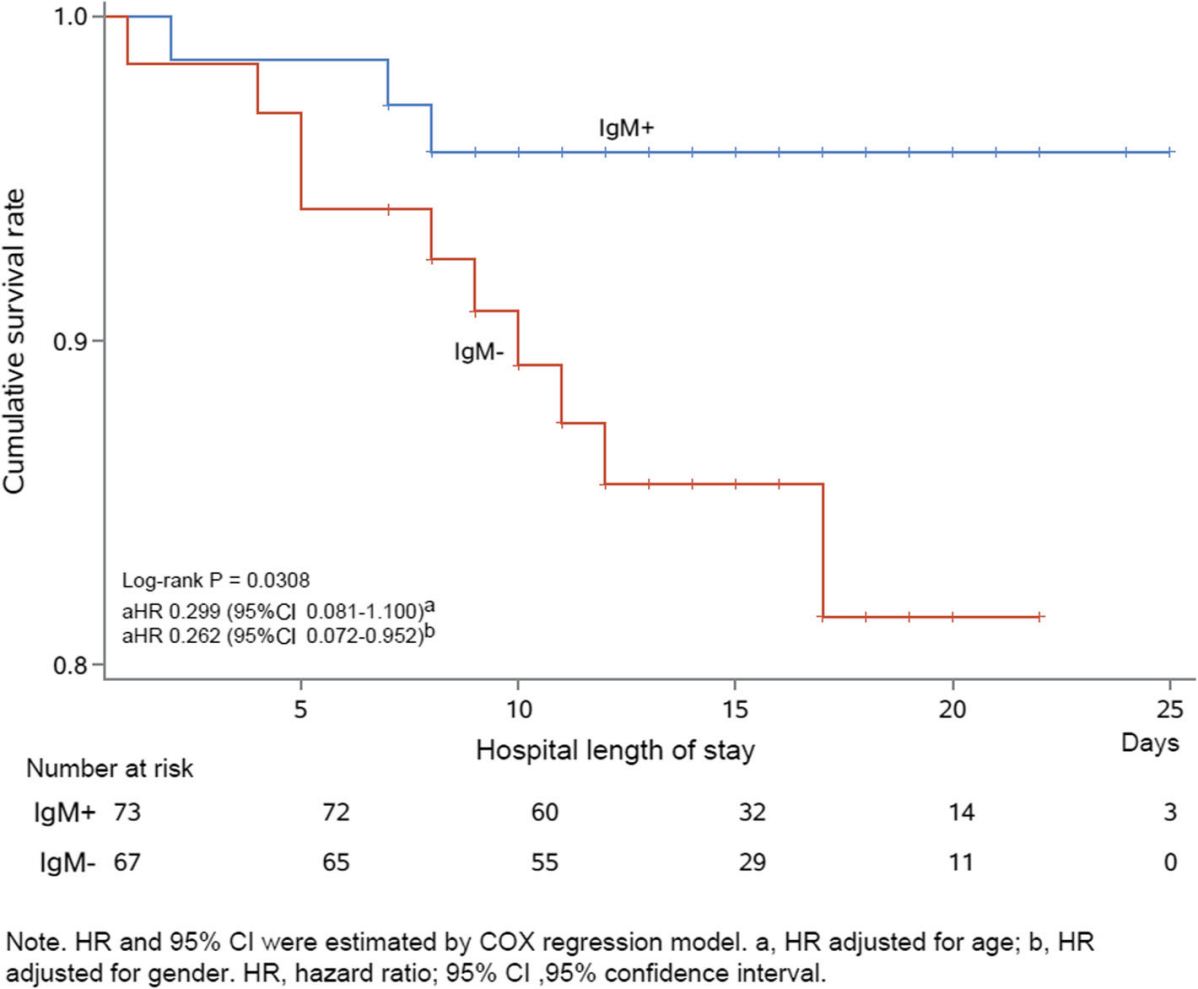
Kaplan-Meier curve for portraying the cumulative survival rate of COVID-19 patients

Severe COVID-19 Patients with influenza virus IgM positive have a higher cumulative survivor rate than that of patients with influenza virus IgM negative (Log-rank *P* = 0.0308). Considering age is a potential confounding variable, difference in age was adjusted between different influenza virus IgM status groups, the HR was 0.29 (95% CI, 0.081–1.100). Similarly, difference in gender was adjusted as above, the HR was 0.262 (95% CI, 0.072–0.952) in the COX regression model. (Fig. 2).

## Discussion

In this retrospective cohort study, we described the clinical features and outcomes of hospitalized COVID-19 patients with different influenza virus IgM status. We found that influenza virus IgM positive may be associated with decreasing in-hospital death. Fatigue and myalgia were less presented in COVID-19 patients with influenza virus IgM positive. It is the first time for influenza virus IgM to be a prognostic factor of COVID-19.

Previous studies reported cases with co-infection of SARS-CoV-2 and influenza showed the implications of co-infection during the pandemic area [17–20]. It was necessary to assess the effect of the SARS-CoV-2 and influenza co-infection in clinical outcomes. Previous studies demonstrated that influenza virus-specific IgM antibody responses follow primary influenza virus infection in adults [11, 21]. Serological confirmation of a clinical diagnosis is by demonstration of greater rise in functional strain specific antibody titer. Specific neutralizing antibody can be detected from about 10 to 14 days post infection, reaches a plateau at around 28 days and decreased to normal level around a month and a half. This test uses nucleocapsid antigens that are type-specific and can distinguish A from B and C infections. Due to the huge task of rapid tests for SARS-CoV-2 and the absence of widely available testing methods, thousands of patients were diagnosed of COVID-19 without identification of co-infection pathogens at the initial period. During the epidemic of seasonal influenza and other respiratory illness, our concern is on the possibility of the co-infection of virus. Therefore, influenza virus IgM antibody may help us review these cases. The outbreak of COVID-19 may occur during influenza season, which brings difficulty in prevention, diagnosis and treatment. Increasing number of literatures has been demonstrating that influenza virus infection may trigger non-neutralizing antibodies responses which also binds to pathogens as diverse as HIV-1, herpes simplex virus and Ebola [22–28]. Some other researches showed that influenza vaccination could reduce cardiovascular morbidity and mortality in patients with COVID-19 [29] Therefore, some potential mechanisms including active immunity or passive immunity may involve in the virus immunity for exhibition its protective effects. In this study, influenza virus IgM positive showed as a protective effector in severe COVID-19 patients associated with better prognosis and higher cumulative survivor rate. Considering the potential confounding variables, age and gender were adjusted between different influenza virus IgM status groups, respectively. After that, the potential protective effects influenza virus IgM positive in severe COVID-19 patients were observed If patients are suspected ILI, especially suffering from virus infection, a prompt test, like a one-time diagnostic panel for the respiratory virus nucleic acid, antigen or serological detection of virus specific IgM/ IgG, should be the first step with an expanded detectable rang towards confirming diagnosis, which help in making early and effective prevention and treatment strategy.

The strengths of this study include adults hospitalized with diagnosis of COVID-19, the retrospective cohort design, standardized patient screening in the participating, and centralized confirmation of respiratory viruses and other laboratory indices. Our study has several limitations. Firstly, a large number of patients were continually being admitted to hospital, but the sample size of our study is still limited. Secondly, our study was conducted in a local hospital in Wuhan, which may result in biases. Especially consideration of influenza season, it may become epidemic of different type in different regions. Thirdly, this cohort study did not last for a long time. Missing information of death status at discharge and initial influenza virus IgM status may influence the demographics and available clinical characteristics between included and excluded patients. Thus, the results may partly help us recognize co-infection of influenza and SARS-CoV-2. Further studies focused on the co-infectious pathogens, the treatment and prevention will be needed.

## Conclusions

Influenza virus IgM positive may be associated with decreasing in-hospital death. The co-infection of SARS-CoV-2 and influenza virus may occur by causing a crisis and we need to improve our understanding for confronting it in the future.

## Supplementary Information

**Additional file 1: Supplementary Table 1**. Clinical Characteristics of COVID-19 Patients by death or discharge

**Additional file 2: Supplementary Table 2**. Clinical Characteristics, treatment and prognosis of COVID-19 Patients by death and influenza IgM+/−

## Data Availability

The data used for the current study will be available based on a reasonable request from the lead author Dr. Yanming Li (lymyl@263.net).

## Abbreviations

APTT: Activated partial thromboplastin time
CDC: Centers for disease control and prevention
COVID-19: Coronavirus disease 2019
ECMO: Extracorporeal membrane oxygenation
eGFR: Estimated glomerular filtration rate
ELISA: Enzyme-linked immunosorbent assay
FiO2: Fraction of inspired oxygen
HA: Hemagglutinin
HFNC: High-flow nasal cannula
ILI: Influenza like illness
IQR: Interquartile range
LDH: Lactate dehydrogenase
MV: Mechanical ventilation
NMV: Noninvasive methods of mechanical ventilation
NT-proBNP: N-terminal pro brain natriuretic peptide
PaO2: Partial pressure of oxygen
PT: Prothrombin time
RT-PCR: Reverse-transcriptase–polymerase chain-reaction
SARS-CoV: Severe acute respiratory syndrome- coronavirus
SARS-CoV-2: Severe acute respiratory syndrome- coronavirus − 2
WHO: World Health Organization
ALT: Alanine transaminase
AST: Aspartate aminotransferase
CRP: C-reactive protein
IL-6: Interleukin-6
FIB: Fibrinogen
NMV: Non-invasive mechanical ventilation
IMV: Invasive mechanical ventilation
