# Supplementary material for: Clinical characteristics and outcome of influenza virus infection among adults hospitalized with severe COVID-19: a retrospective cohort study from Wuhan, China
Source: BMC Infect Dis. 2021 Apr 12;21:341. doi: 10.1186/s12879-021-05975-2 (PMC8040362; doi:10.1186/s12879-021-05975-2)
Supplement: Supplementary file 1 — Additional file 1: Supplementary Table 1. Clinical Characteristics of COVID-19 Patients by death or discharge [file 12879_2021_5975_MOESM1_ESM.docx]

**Supplementary Table 1. Clinical Characteristics of COVID-19 Patients by death or discharge**

| **Study Population** | **Death (n=13)** | **Discharge (n=127)** | **Total (n=140)** | **P value** |
| --- | --- | --- | --- | --- |
| Demographic |  |  |  |  |
| Gender, Male | 10 (76.9) | 66 (52.0) | 76 (54.3) | 0.0854 |
| Age, media (IQR), yrs | 64.0 (56.0, 71.0) | 65.0 (48.0, 70.0) | 65.0 (48.5, 70.0) | 0.3673 |
| Comorbidities |  |  |  |  |
| Chronic respiratory disease | 0/13 (0.0) | 10/126 (7.9) | 10/139 (7.2) | 0.5981 |
| Malignancy | 2/13 (15.4) | 4/126 (3.2) | 6/139 (4.3) | 0.0980 |
| Hypertension | 4/13 (30.8) | 58/124 (46.8) | 62/137 (45.3) | 0.2700 |
| Diabetes | 2/13 (15.4) | 20/126 (15.9) | 22/139 (15.8) | 0.9632 |
| Cardiovascular disease | 1/13 (7.7) | 7/126 (5.6) | 8/139 (5.8) | 0.5539 |
| Chronic kidney disease | 0/13 (0.0) | 3/126 (2.4) | 3/139 (2.2) | 1.0000 |
| Signs and symptoms |  |  |  |  |
| Fever | 12 (92.3) | 96 (75.6) | 108 (77.1) | 0.1294 |
| Highest temperature, °C | 38.8 (38.2, 39.0) | 38.5 (38.0, 39.0) | 38.5 (38.0, 39.0) | 0.4296 |
| Chills | 2 (15.4) | 30 (23.6) | 32 (22.9) | 0.4829 |
| Cough | 8/13 (61.5) | 77/126 (61.1) | 85/139 (61.2) | 0.9760 |
| Productive cough | 4/13 (30.8) | 41/126 (32.5) | 45/139 (32.4) | 0.8962 |
| Chest pain/Chest congestion | 3/13 (23.1) | 29/126 (23.0) | 32/139 (23.0) | 0.9960 |
| Dyspnea | 6/13 (46.2) | 44/126 (34.9) | 50/139 (36.0) | 0.4285 |
| Diarrhea | 4 (30.8) | 39 (30.7) | 43 (30.7) | 0.9964 |
| Fatigue or myalgia | 6/13 (46.2) | 52/126 (41.3) | 58/139 (41.7) | 0.7339 |
| Laboratory findings, median (IQR) |  |  |  |  |
| White blood cells, ×10^9^/mL | 6.7 (4.6, 8.5) | 5.6 (4.4, 7.2) | 5.7 (4.4, 7.2) | 0.4137 |
| Neutrophils, ×10^9^/mL | 5.6 (2.7, 7.9) | 3.8 (2.5, 5.0) | 3.9 (2.5, 5.3) | 0.1880 |
| Lymphocytes, ×10^9^/mL | 0.5 (0.4, 0.9) | 1.2 (0.9, 1.6) | 1.1 (0.8, 1.5) | 0.0004 |
| Lymphocytes<0.8×10^9^/mL | 9/13 (69.2) | 27/126 (21.4) | 36/139 (25.9) | 0.0005 |
| Red blood cells, ×1012/mL | 3.6 (2.4, 4.5) | 4.1 (3.7, 4.4) | 4.0 (3.7, 4.5) | 0.1148 |
| Platelets, ×10^9^/ mL | 145.0 (83.0, 222.0) | 240.5 (184.0, 321.0) | 235.0 (169.0, 312.0) | 0.0102 |
| Platelets <100×10^9^/mL | 4/13 (30.8) | 7/126 (5.6) | 11/139 (7.9) | 0.0092 |
| Hemoglobin, g/L | 107.0 (89.0, 139.0) | 124.5 (115.0, 137.0) | 123.0 (113.0, 137.0) | 0.1418 |
| ALT, U/L | 19.0 (18.0, 37.0) | 24.0 (15.0, 41.0) | 23.0 (16.0, 41.0) | 0.5898 |
| AST, U/L | 46.0 (38.0, 53.0) | 26.5 (19.0, 37.0) | 28.0 (19.0, 39.0) | 0.0037 |
| Albumin, g/L | 34.0 (29.6, 36.2) | 35.2 (32.1, 38.1) | 35.2 (31.7, 38.1) | 0.0864 |
| Creatinine, μmol/L | 98.0 (78.0, 114.0) | 69.0 (59.0, 84.0) | 70.0 (59.0, 89.0) | 0.0238 |
| LDH, U/L | 375.5 (302.0, 589.5) | 275.0 (209.0, 329.0) | 281.0 (212.0, 334.0) | 0.0039 |
| LDH > 245 U/L | 11/12 (91.7) | 80/125 (64.0) | 91/137 (66.4) | 0.0315 |
| Troponin >15.6pg/mL, No (%) | 9/13 (69.2) | 10/94 (10.6) | 19/107 (17.8) | <.0001 |
| NT-proBNP, pg/mL | 549.0 (416.5, 942.0) | 124.0 (61.0, 314.0) | 151.0 (63.0, 411.0) | 0.0005 |
| NT-proBNP ≥247pg/mL, No (%) | 11/12 (91.7) | 53/103 (51.5) | 64/115 (55.7) | 0.0080 |
| CRP, mg/L | 60.6 (42.6, 163.4) | 22.6 (5.3, 51.2) | 27.2 (6.1, 69.8) | 0.0013 |
| CRP ≥1mg/L, No (%) | 13/13 (100.0) | 91/97 (93.8) | 104/110 (94.5) | 1.0000 |
| IL-6, pg/mL | 35.6 (23.2, 57.5) | 6.9 (3.6, 19.6) | 9.4 (3.9, 23.2) | 0.0052 |
| IL-6 ≥7pg/mL, No (%) | 6/6 (100.0) | 34/71 (47.9) | 40/77 (51.9) | 0.0039 |
| Ferritin, μg/L | 1581.8 (563.1, 2964.8) | 547.5 (320.5, 828.4) | 562.6 (320.5, 986.5) | 0.0160 |
| Ferritin >150μg/L, No (%) | 10/12 (83.3) | 62/66 (93.9) | 72/78 (92.3) | 0.2287 |
| PT, s | 14.7 (13.7, 15.4) | 13.7 (13.3, 14.2) | 13.8 (13.3, 14.3) | 0.0188 |
| APTT, s | 45.4 (38.2, 46.2) | 39.4 (36.6, 43.3) | 39.6 (36.6, 44.3) | 0.1641 |
| APTT >42s, No (%) | 8/13 (61.5) | 37/123 (30.1) | 45/136 (33.1) | 0.0268 |
| FIB, g/L | 4.9 (3.9, 5.9) | 5.0 (4.1, 6.1) | 5.0 (4.1, 6.1) | 0.5864 |
| D-Dimer, μg/mL | 2.0 (1.0, 3.0) | 0.8 (0.5, 1.8) | 1.0 (0.5, 2.0) | 0.0270 |
| D-Dimer≥0.5μg/mL, No (%) | 11/13 (84.6) | 87/124 (70.2) | 98/137 (71.5) | 0.2458 |
| Treatment in hospital |  |  |  |  |
| Oxygen Therapy |  |  |  |  |
| Nasal Cannula | 0 (0.0) | 61 (48.0) | 61 (43.6) | 0.0009 |
| Oxygen Mask | 1 (7.7) | 2 (1.6) | 3 (2.1) | 0.2552 |
| NMV/High-flow nasal cannula | 5 (38.5) | 64 (50.4) | 69 (49.3) | 0.4124 |
| IMV/ECMO | 7 (53.8) | 5 (3.9) | 12 (8.6) | <.0001 |
| Drugs |  |  |  |  |
| Oseltamivir | 3 (23.1) | 53 (41.7) | 56 (40.0) | 0.1910 |
| Arbidol | 8 (61.5) | 92 (72.4) | 100 (71.4) | 0.4193 |
| Compound Methoxamine capsule | 0 (0.0) | 23 (18.1) | 23 (16.4) | 0.0265 |
| Clinical outcomes |  |  |  |  |
| CURB-65 |  |  |  | <.0001 |
| Low risk | 1 (7.7) | 114 (89.8) | 115 (82.1) |  |
| Medium risk | 1 (7.7) | 13 (10.2) | 14 (10.0) |  |
| High risk | 11 (84.6) | 0 (0.0) | 11 (7.9) |  |
| Duration of viral shedding, days | 34.0 (34.0, 34.0) | 25.0 (20.0, 32.0) | 25.5 (20.5, 32.0) | 0.3033 |
| Hospital length of stay, days | 8.0 (5.0, 10.0) | 14.0 (11.0, 18.0) | 13.0 (10.0, 18.0) | <.0001 |
| Time from illness onset to discharge, days | 18.0 (13.0, 29.0) | 27.0 (23.0, 34.0) | 27.0 (22.0, 33.5) | 0.0078 |

Note. Data are presented as n (%) or median (IQR, interquartile range) for each parameter. *P* values were calculated by chi-square test, Fisher’s exact test, or Mann-Whitney U test, where appropriate.

Abbreviations. IQR, interquartile range; ALT, alanine transaminase; AST, aspartate aminotransferase; LDH, lactic Acid dehydrogenase; CRP, C-reactive protein; IL-6, interleukin-6; PT, prothrombintime; APTT, activated partial thromboplastin time; FIB, fibrinogen; NMV, non-invasive mechanical ventilation; IMV, invasive mechanical ventilation; ECMO, extracorporeal membrane oxygenation.
