# Supplementary material for: Clinical characteristics and outcome of influenza virus infection among adults hospitalized with severe COVID-19: a retrospective cohort study from Wuhan, China
Source: BMC Infect Dis. 2021 Apr 12;21:341. doi: 10.1186/s12879-021-05975-2 (PMC8040362; doi:10.1186/s12879-021-05975-2)
Supplement: Supplementary file 2 — Additional file 2: Supplementary Table 2. Clinical Characteristics, treatment and prognosis of COVID-19 Patients by death and influenza IgM+/− [file 12879_2021_5975_MOESM2_ESM.docx]

**Supplementary Table 2. Clinical Characteristics, treatment and prognosis of COVID-19 Patients by death and influenza IgM+/-**

| **Study Population** | **Discharge & IgM- (n=57)** | **death & IgM- (n=10)** | **Discharge & IgM+ (n=70)** | **death & IgM+ (n=3)** | **Total (n=140)** |
| --- | --- | --- | --- | --- | --- |
| Demographic |  |  |  |  |  |
| Gender, Male | 28 (49.1) | 9 (90.0) | 38 (54.3) | 1 (33.3) | 76 (54.3) |
| Age, media (IQR), yrs | 66.0 (56.0, 72.0) | 60.5 (55.0, 68.0) | 61.5 (46.0, 68.0) | 88.0 (71.0, 88.0) | 65.0 (48.5, 70.0) |
| Comorbidities |  |  |  |  |  |
| Chronic respiratory disease | 5/57 (8.8) | 0/10 (0.0) | 5/69 (7.2) | 0/3 (0.0) | 10/139 (7.2) |
| Malignancy | 1/57 (1.8) | 2/10 (20.0) | 3/69 (4.3) | 0/3 (0.0) | 6/139 (4.3) |
| Hypertension | 27/57 (47.4) | 3/10 (30.0) | 31/67 (46.3) | 1/3 (33.3) | 62/137 (45.3) |
| Diabetes | 9/57 (15.8) | 1/10 (10.0) | 11/69 (15.9) | 1/3 (33.3) | 22/139 (15.8) |
| Cardiovascular disease | 2/57 (3.5) | 1/10 (10.0) | 5/69 (7.2) | 0/3 (0.0) | 8/139 (5.8) |
| Chronic kidney disease | 1/57 (1.8) | 0/10 (0.0) | 2/69 (2.9) | 0/3 (0.0) | 3/139 (2.2) |
| Signs and symptoms |  |  |  |  |  |
| Fever | 43 (75.4) | 10 (100.0) | 53 (75.7) | 2 (66.7) | 108 (77.1) |
| Highest temperature, °C | 38.7 (38.0, 39.0) | 38.7 (38.2, 39.0) | 38.5 (38.0, 39.0) | 38.9 (38.9, 38.9) | 38.5 (38.0, 39.0) |
| Chills | 17 (29.8) | 2 (20.0) | 13 (18.6) | 0 (0.0) | 32 (22.9) |
| Cough | 37/57 (64.9) | 7/10 (70.0) | 40/69 (58.0) | 1/3 (33.3) | 85/139 (61.2) |
| Productive cough | 22/57 (38.6) | 3/10 (30.0) | 19/69 (27.5) | 1/3 (33.3) | 45/139 (32.4) |
| Chest pain/Chest congestion | 11/57 (19.3) | 2/10 (20.0) | 18/69 (26.1) | 1/3 (33.3) | 32/139 (23.0) |
| Dyspnea | 24/57 (42.1) | 5/10 (50.0) | 20/69 (29.0) | 1/3 (33.3) | 50/139 (36.0) |
| Diarrhea | 21 (36.8) | 4 (40.0) | 18 (25.7) | 0 (0.0) | 43 (30.7) |
| Fatigue or myalgia | 29/57 (50.9) | 5/10 (50.0) | 23/69 (33.3) | 1/3 (33.3) | 58/139 (41.7) |
| Laboratory findings, median (IQR) |  |  |  |  |  |
| White blood cells, ×10^9^/mL | 5.7 (4.6, 7.8) | 5.9 (4.6, 10.3) | 5.6 (4.2, 6.8) | 8.4 (3.3, 8.5) | 5.7 (4.4, 7.2) |
| Neutrophils, ×10^9^/mL | 3.8 (2.6, 5.8) | 4.9 (2.3, 8.9) | 3.9 (2.5, 4.8) | 6.6 (2.7, 7.9) | 3.9 (2.5, 5.3) |
| Lymphocytes, ×10^9^/mL | 1.1 (0.9, 1.6) | 0.5 (0.3, 0.9) | 1.2 (0.9, 1.6) | 0.5 (0.4, 1.3) | 1.1 (0.8, 1.5) |
| Lymphocytes<0.8×10^9^/mL | 11/56 (19.6) | 7/10 (70.0) | 16/70 (22.9) | 2/3 (66.7) | 36/139 (25.9) |
| Red blood cells, ×10^12^/mL | 4.1 (3.8, 4.4) | 3.6 (2.2, 4.5) | 4.1 (3.7, 4.6) | 3.6 (3.3, 5.1) | 4.0 (3.7, 4.5) |
| Platelets, ×10^9^/ mL | 262.0 (196.0, 350.5) | 151.0 (69.0, 222.0) | 230.5 (179.0, 292.0) | 106.0 (83.0, 342.0) | 235.0 (169.0, 312.0) |
| Platelets<100×10^9^/mL | 3/56 (5.4) | 3/10 (30.0) | 4/70 (5.7) | 1/3 (33.3) | 11/139 (7.9) |
| Hemoglobin, g/L | 126.5 (115.5, 135.0) | 107.0 (65.0, 139.0) | 122.0 (114.0, 137.0) | 114.0 (89.0, 152.0) | 123.0 (113.0, 137.0) |
| ALT, U/L | 23.5 (15.0, 41.5) | 19.5 (17.0, 37.0) | 24.5 (16.0, 41.0) | 19.0 (18.0, 22.0) | 23.0 (16.0, 41.0) |
| AST, U/L | 27.0 (19.0, 37.0) | 44.5 (38.0, 59.0) | 26.0 (19.0, 37.0) | 46.0 (20.0, 53.0) | 28.0 (19.0, 39.0) |
| Albumin, g/L | 35.2 (31.9, 37.5) | 32.8 (29.6, 35.4) | 35.7 (32.2, 38.3) | 36.3 (23.5, 38.9) | 35.2 (31.7, 38.1) |
| Creatinine, μmol/L | 69.5 (59.0, 82.5) | 92.5 (53.0, 114.0) | 69.0 (60.0, 86.0) | 98.0 (91.0, 148.0) | 70.0 (59.0, 89.0) |
| LDH, U/L | 280.0 (232.0, 325.5) | 411.0 (351.0, 674.0) | 261.0 (202.0, 330.0) | 302.0 (286.0, 314.0) | 281.0 (212.0, 334.0) |
| LDH > 245 U/L | 39/56 (69.6) | 8/9 (88.9) | 41/69 (59.4) | 3/3 (100.0) | 91/137 (66.4) |
| Troponin >15.6 pg/mL, No (%) | 6/46 (13.0) | 6/10 (60.0) | 4/48 (8.3) | 3/3 (100.0) | 19/107 (17.8) |
| NT-proBNP, pg/mL | 124.0 (61.0, 335.0) | 549.0 (350.0, 995.0) | 124.0 (57.0, 314.0) | 698.0 (507.0, 889.0) | 151.0 (63.0, 411.0) |
| NT-proBNP ≥247pg/mL, No (%) | 26/48 (54.2) | 9/10 (90.0) | 27/55 (49.1) | 2/2 (100.0) | 64/115 (55.7) |
| CRP, mg/L | 27.5 (6.5, 70.4) | 59.6 (40.2, 165.4) | 20.1 (3.9, 45.0) | 84.1 (51.3, 163.4) | 27.2 (6.1, 69.8) |
| CRP≥1mg/L, No (%) | 37/39 (94.9) | 10/10 (100.0) | 54/58 (93.1) | 3/3 (100.0) | 104/110 (94.5) |
| IL-6, pg/mL | 6.0 (3.5, 17.3) | 35.6 (26.4, 49.5) | 9.6 (4.1, 20.6) | 39.9 (14.5, 65.3) | 9.4 (3.9, 23.2) |
| IL-6 ≥ 7pg/mL, No (%) | 11/31 (35.5) | 4/4 (100.0) | 23/40 (57.5) | 2/2 (100.0) | 40/77 (51.9) |
| Ferritin, μg/L | 595.8 (310.2, 903.0) | 1611.8 (892.5, 3507.4) | 517.7 (324.5, 666.3) | 1551.7 (88.1, 1968.1) | 562.6 (320.5, 986.5) |
| Ferritin>150μg/L, No (%) | 25/26 (96.2) | 8/9 (88.9) | 37/40 (92.5) | 2/3 (66.7) | 72/78 (92.3) |
| PT, s | 13.8 (13.4, 14.2) | 14.0 (13.5, 16.0) | 13.7 (13.2, 14.3) | 15.0 (14.9, 15.4) | 13.8 (13.3, 14.3) |
| APTT, s | 39.3 (37.5, 45.1) | 45.7 (39.6, 48.1) | 39.7 (35.9, 42.0) | 34.0 (29.7, 45.4) | 39.6 (36.6, 44.3) |
| APTT>42s, No (%) | 21/54 (38.9) | 7/10 (70.0) | 16/69 (23.2) | 1/3 (33.3) | 45/136 (33.1) |
| FIB, g/L | 5.3 (4.3, 6.2) | 5.1 (3.0, 6.3) | 4.9 (3.8, 6.1) | 4.1 (3.9, 5.7) | 5.0 (4.1, 6.1) |
| D-Dimer, μg/mL | 1.2 (0.5, 2.0) | 1.7 (0.6, 2.6) | 0.7 (0.5, 1.6) | 3.0 (2.0, 17.7) | 1.0 (0.5, 2.0) |
| D-Dimer≥0.5μg/mL, No (%) | 37/54 (68.5) | 8/10 (80.0) | 50/70 (71.4) | 3/3 (100.0) | 98/137 (71.5) |
| Treatment in hospital |  |  |  |  |  |
| Oxygen Therapy |  |  |  |  |  |
| Nasal Cannula | 29 (50.9) | 0 (0.0) | 32 (45.7) | 0 (0.0) | 61 (43.6) |
| Oxygen Mask | 1 (1.8) | 1 (10.0) | 1 (1.4) | 0 (0.0) | 3 (2.1) |
| NMV/High-flow nasal cannula | 30 (52.6) | 4 (40.0) | 34 (48.6) | 1 (33.3) | 69 (49.3) |
| IMV/ECMO | 0 (0.0) | 5 (50.0) | 5 (7.1) | 2 (66.7) | 12 (8.6) |
| Drugs |  |  |  |  |  |
| Oseltamivir | 20 (35.1) | 3 (30.0) | 33 (47.1) | 0 (0.0) | 56 (40.0) |
| Arbidol | 41 (71.9) | 6 (60.0) | 51 (72.9) | 2 (66.7) | 100 (71.4) |
| Compound Methoxamine capsule | 6 (10.5) | 0 (0.0) | 17 (24.3) | 0 (0.0) | 23 (16.4) |
| Clinical outcomes |  |  |  |  |  |
| CURB-65 |  |  |  |  |  |
| Low risk | 49 (86.0) | 1 (10.0) | 65 (92.9) | 0 (0.0) | 115 (82.1) |
| Medium risk | 8 (14.0) | 0 (0.0) | 5 (7.1) | 1 (33.3) | 14 (10.0) |
| High risk | 0 (0.0) | 9 (90.0) | 0 (0.0) | 2 (66.7) | 11 (7.9) |
| Duration of viral shedding, days | 25.0 (21.0, 31.0) | 34.0 (34.0, 34.0) | 26.0 (20.0, 32.0) | - | 25.5 (20.5, 32.0) |
| Hospital length of stay, days | 14.0 (12.0, 18.0) | 8.5 (5.0, 11.0) | 13.0 (11.0, 18.0) | 7.0 (2.0, 8.0) | 13.0 (10.0, 18.0) |
| Time from illness onset to discharge, days | 27.0 (23.0, 33.0) | 19.5 (13.0, 33.0) | 27.5 (22.0, 35.0) | 13.0 (8.0, 29.0) | 27.0 (22.0, 33.5) |

Abbreviations. IQR, interquartile range; ALT, alanine transaminase; AST, aspartate aminotransferase; LDH, lactic Acid dehydrogenase; CRP, C-reactive protein; IL-6, interleukin-6; PT, prothrombintime; APTT, activated partial thromboplastin time; FIB, fibrinogen; NMV, non-invasive mechanical ventilation; IMV, invasive mechanical ventilation; ECMO, extracorporeal membrane oxygenation.
